# Effect of homemade peanut oil consumption during pregnancy on low birth weight and preterm birth outcomes: a cohort study in Southwestern China

**DOI:** 10.1080/16549716.2024.2336312

**Published:** 2024-04-17

**Authors:** Yanxu Zhong, Huan Lu, Yuyan Jiang, Minyan Rong, Xiangming Zhang, Tippawan Liabsuetrakul

**Affiliations:** a Food Safety Monitoring and Evaluation Department, Guangxi Zhuang Autonomous Region Centre for Disease Control and Prevention (Guangxi CDC), Nanning, China; b Department of Epidemiology, Faculty of Medicine, Prince of Songkla University, Hat Yai, Songkhla, Thailand; c Infectious Diseases Department, The Fourth People’s Hospital of Nanning, Nanning, China; d Infectious Disease Control and Prevention Department, Guiping Center for Disease Control and Prevention (Guiping CDC), Guigang, China; e Maternity Department, Guiping People’s Hospital, Guigang, China

**Keywords:** Aflatoxins, homemade peanut oil, low birth weight, preterm birth, cohort study, logistic regression

## Abstract

**Background:**

Homemade peanut oil is widely consumed in rural areas of Southwestern China, which is easily contaminated by aflatoxins (AFs) and associated with adverse birth outcomes.

**Objective:**

To identify the effect of exposure to homemade peanut oil consumption on low birth weight (LBW), preterm birth (PB) and other associated factors.

**Methods:**

A prospective cohort study was conducted among pregnant women in Guangxi province, Southwestern China. Information of all eligible women on homemade peanut oil consumption and potential factors associated with LBW and PB was collected, and all were followed up until delivery. The effect of homemade peanut oil exposure was analyzed using multiple logistic regression models using the directed acyclic graph (DAG) approach.

**Results:**

Of 1611 pregnant women, 1316 (81.7%) had consumed homemade peanut oil, and the rates of LBW and PB were 9.7% and 10.0%, respectively. Increased risks of LBW and PB in women with homemade peanut oil consumption were found with aORs of 1.9 (95% CI 1.1–3.2) and 1.8 (95% CI 1.1–3.0), respectively. Women with a history of PB or LBW were 3–5 times more likely to have higher rates of LBW or PB compared with those without this type of history. The odds of PB were approximately double in those taking medicine during pregnancy. Advanced maternal age, lack of physical exercise during pregnancy, passive smoking, or pregnancy complications were also more likely to have a higher risk of LBW.

**Conclusions:**

Homemade peanut oil consumption was a potential risk factor for both LBW and PB, of which health authorities who are responsible for food safety of the country should pay more attention to providing recommendation for oil consumption during pregnancy.

## Paper Context

- **Main findings:** Homemade peanut oil consumption was associated with increased risk of low birth weight and preterm birth, in addition to advanced age, adverse obstetric histories, and health risk behaviors during pregnancy in a county in Southwestern China.
- **Added knowledge:** This study identifies the direct and total effects of homemade peanut oil consumption on low birth weight and preterm birth and explains the factors associated with low birth weight and preterm birth in a county in Southwestern China.
- **Global health impact for policy and action:** Evidence of associated risk factors for low birth weight and preterm birth should be informed to the community, and precautionary policies for the protection of aflatoxin exposure during pregnancy are needed.

## Background

Aflatoxins (AFs) are commonly found in contaminated staple foods such as maize, nuts, peanuts, corn, and oils, leading to chronic exposure in humans [1] which are shown to be associated with carcinogenicity and genotoxicity [2]. Maternal immunity and fetal growth can also be impaired when pregnant women are exposed to AFs [3–5]. Homemade peanut oil is made naturally by peanut-squeezing machines in households or small-scale oil mills, which can be contaminated by AFs. Homemade peanut oil has a lower price and a special taste and has been the main cooking oil in the southwestern rural area of China [6,7], such as rural areas of Guangxi province; however, the rate of homemade peanut oil use has not been locally and nationally studied. A previous study in Guangxi reported that aflatoxin B1 (AFB1), the most toxic and frequent type of AF, was detected in 78.1% of oil samples [8]. Health risks of cancer from dietary exposure AFs in China, not fetal growth effects, were only studied [8,9], although a study conducted in Gambia showed the association of AFs and fetal weights [10].

The World Health Organization defines low birth weight (LBW) as a birth weight below 2500 g regardless of gestational age, and preterm birth (PB) as neonate born before 37 completed weeks of gestation [11]. The rates of LBW and PB were globally estimated at 14.6% of live births in 2015 [12] and 10.6% of live births in 2014 [13], respectively, of which the majority occurred in low-income and middle-income countries. LBW is an important factor of neonatal mortality and morbidity and is closely associated with short and long‐term consequences [14]. Likewise, the PB was the second most common cause of death in children aged under 5 years in the world [15]. A systematic review, involving the studies mostly from the United States, identified considerable long-term economic impact of low birth weight and preterm birth [16].

A previous study in Guiping county, Guangxi in Southwestern China found higher prevalence of LBW (10.4%) and PB (11.1%) [17] compared to the national average rates of LBW (6.1%) and PB (5.7%) in China [18]. Most residents living in Guiping county consume homemade peanut oil. However, there have been no studies to date examining whether LBW or PB are related to the consumption of homemade peanut oil. Based on the high consumption of homemade peanut oil and higher prevalence of LBW and PB in Guiping country, our study aimed to examine the effect of consumption of homemade peanut oil during pregnancy on LBW and PB adjusted for confounders and explore other independent risk factors for LBW and PB.

## Materials and methods

### Study design and study setting

A prospective cohort study was conducted in Guiping county, Southwestern China, during December 2021-May 2022. Pregnant women of at least 28 weeks of gestation living in Guiping county who come for antenatal care and planned to give birth at the study hospital were included. A gestational age of 28 weeks was used based on the definition of live birth in accordance with the definition of stillbirth defined from the World Health Organization [19]. In this county, a county hospital where both services of antenatal and labor/childbirth care are available with the highest records of delivery, approximately 4000 each year, was chosen to be the study hospital. Those with multiple pregnancies and those having known medical diseases, such as hypertension, diabetes, autoimmune diseases, and neurological disorders before pregnancy due to well-known risk of LBW and PB, or verbal communication disorders, such as dumbness, deafness, or mental retardation, were excluded.

### Study sample size and participants

We used a formula for the difference in the two proportions of LBW birth outcomes between homemade peanut oil and non-homemade peanut oil consumption to be at 5% (10.5% vs 5.5%). Because the percentage of residents in Guiping who consumed homemade peanut nut oil was 80%, we set the ratio of exposure and non-exposure to homemade peanut oil at 4:1. Given a type I error of 5%, power of 80% and a ratio of 4, at least 291 non-homemade and 1164 homemade peanut oil participants were required.

**Figure 1.**
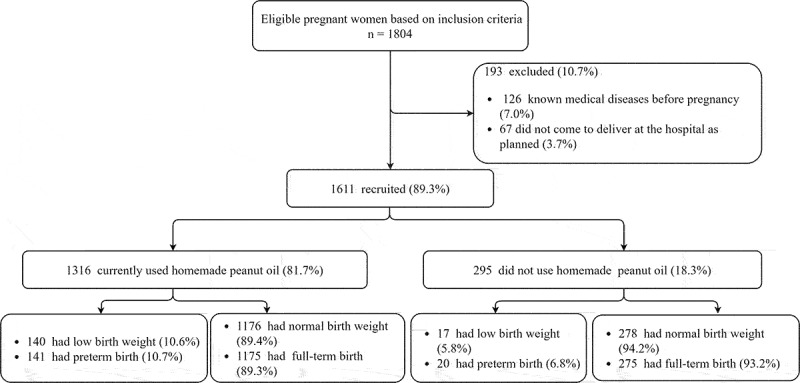
Study flow diagram of the cohort study.

Figure 1 shows the flow diagram for study recruitment. A total of 1804 participants met the inclusion criteria during the study period, of which 126 (7.0%) were excluded due to having unknown medical diseases before pregnancy and 67 women did not come to deliver at the setting hospital as planned (3.7%). Finally, the data of 1611 participants were included in the analysis, of whom 1316 (81.7%) were in the homemade peanut oil group and 295 (18.3%) were in the non-homemade peanut oil group.

### Variables

The main outcomes of this study were LBW and PB, based on the WHO definitions. Low birth weight was defined as a newborn weight below 2500 g (yes, no). Preterm birth was defined as a baby born alive before 37 complete weeks of gestation but after 28 weeks of gestation (yes, no). The main exposure of interest was consumption of either homemade or non-homemade peanut oil. Since there is no standard threshold for recommended consumption of homemade peanut oil available, in cases where a woman consumed both types of oil (*n* = 30, 1.9%), we used the consumption at least 60% of the time to be grouped into homemade peanut oil.

The demographic characteristics included age group, ethnicity, residency, education level, current occupation, and family income. Age groups were calculated from age (years) and categorized into <20, 20–34, ≥35 [18]. Ethnicity was categorized into Han, Zhuang, and others. Residency was categorized as urban and rural. Education level was categorized as middle school and below, high school, college and above. Current occupation was categorized as government staff, farmer or worker, self-employed, and others. Family income was converted from Chinese Yuan (CNY) into United States Dollars (USD) and categorized as ≤450 USD/month, 450–750 USD/month, 750–1500 USD/month, >1500 USD/month according to the average income of Guangxi residents in 2020 [20].

The behavior and obstetric information included physical exercise during pregnancy, drinking history, smoking history, passive smoking, history of PB, history of LBW, history of abortion, history of hepatitis B virus (HBV), parity, body mass index (BMI) before pregnancy (pre-pregnancy BMI), pregnancy complications, folic supplementation during pregnancy, calcium supplementation during pregnancy, iron supplementation during pregnancy, vitamin D supplementation during pregnancy, and medicine intake during pregnancy. All the variables were categorized into yes and no, except passive smoking, parity, and pre-pregnancy BMI. Passive smoking was categorized into no, workplace or home, or workplace and home, and parity was categorized into 1, 2 and ≥3. Pre-pregnancy BMIs were calculated by pre-pregnancy weight (kg) divided by height in meters squared and categorized into underweight (<18.5 kg/m^2^), normal (18.5–22.9 kg/m^2^), and overweight or obese (≥23 kg/m^2^) [21].

The birth outcome variables included birth gestation age (weeks), mode of delivery, newborn sex, and weight of newborn (g). The birth gestation age was determined according to the mother’s last menstrual cycle, with ultrasound used to estimate the gestational age if the menstrual date was uncertain. Modes of delivery were categorized into vaginal delivery, cesarean section or assisted vaginal delivery. Newborn sex was categorized as male and female. The weight of the newborn was the weight when the newborn was delivered. All of these variables were collected from the Guangxi maternal and child healthcare system.

### Data collection

All eligible pregnant women were invited to participate in the study, and after signing the information sheet and consent form, they were interviewed using a structured questionnaire and a pregnant woman’s health and antenatal care (ANC) questionnaire. We followed the study of pregnant women until they delivered and collected the newborn-related factors and examination data from the Guangxi maternal and child healthcare system. The questionnaires were checked by investigators by days, and all unconfirmed or missing data were recollected the next day or double-checked through Guangxi maternal and child healthcare system.

### Statistical analysis

All analyses were conducted using R software version 4.1.2 (The R Foundation for Statistical Computing 2020, Vienna, Austria). The characteristics of the participants for continuous data were descriptively analyzed using means ± standard deviations or median, with interquartile range depending on the normal distribution of data or percentages for the categorical data. A directed acyclic graph (DAG) diagram was used to represent the causal relationships between homemade peanut oil consumption and LBW and PB outcomes based on direct and total effects [22] regarding the associated factors obtained from a previous systematic review [23] and additional exploratory factors from a univariate analysis of our study. The direct effect estimates of the causal pathways considered only confounders, not mediators, while the total effect estimates considered both confounders and mediators [24]. The factors used to construct DAGs were the main exposures and outcomes as well as the factors with a *p* value lower than 0.05 in univariate analysis. The effects of homemade peanut oil consumption on LBW and PB in DAGs were analyzed using multiple logistic regression and presented in terms of crude odds ratios (cOR) and adjusted odds ratios (aOR) with 95% confidence intervals (CIs) in both direct and total effects. Apart from the DAG models, other independent risk factors were also analyzed using a multiple logistic regression model considering factors with a *p* value less than 0.2 in univariate analysis included in the first model with a backward stepwise approach using the lowest Akaike Information Criterion (AIC) value. A two-sided *p* < 0.05 was considered statistically significant.

## Results

Their demographic characteristics, divided by consumption or not of using peanut oil, are shown in Table 1. Those living in rural areas, education of middle school or below, working as farmer or worker, and family income less than 450 US$/month were significantly higher in the homemade peanut oil group than in the non-homemade peanut oil group. Behavioral factors and obstetric information of the women between homemade peanut oil and non-homemade peanut oil groups are presented in Table 2. Most behavioral factors and obstetric information between the two groups were not significantly different, except for parity, pre-pregnancy BMI, and folic supplementation, for which primiparous women, women with pre-pregnancy BMI > 23, and those who had received folic supplementation were more likely to be in the homemade peanut oil group.

**Table 1.** Demographic characteristics of study women who currently consumed and did not consume homemade peanut oil.

Variable	Total (*N* = 1611)	Homemade peanut oil (*N* = 1316)	Non-homemade peanut oil (*N* = 295)	*p*
Age group				0.070
<20	64 (4.0)	56 (4.3)	8 (2.7)	
20–34	1229 (76.3)	989 (75.2)	240 (81.4)	
35 or above	318 (19.7)	271 (20.6)	47 (15.9)	
Ethnicity				0.939
Han	1458 (90.5)	1190 (90.4)	268 (90.9)	
Zhuang	42 (2.6)	92 (7.0)	19 (6.4)	
Others	111 (6.9)	34 (2.6)	8 (2.7)	
Residency				0.002
Urban	149 (9.2)	107 (8.1)	42 (14.2)	
Rural	1462 (90.8)	1209 (91.9)	253 (85.8)	
Education				0.003
Middle school and below	844 (52.4)	713 (54.2)	131 (44.4)	
High school	590 (36.6)	471 (35.8)	119 (40.3)	
College and above	177 (11.0)	132 (10.0)	45 (15.3)	
Occupation				<0.001
Government staff	230 (14.3)	175 (13.3)	55 (18.6)	
Famer or worker	711 (44.1)	627 (47.6)	84 (28.5)	
Self-employed	246 (15.3)	197 (15.0)	49 (16.6)	
Others	424 (26.3)	317 (24.1)	107 (36.3)	
Family income (USD/month)				<0.001
<450	196 (12.2)	173 (13.2)	23 (7.8)	
450–750	383 (23.8)	333 (25.3)	50 (16.9)	
751–1500	803 (49.8)	624 (47.4)	179 (60.7)	
>1500	229 (14.2)	186 (14.1)	43 (14.6)

**Table 2.** Behavioral factors and obstetric information of study women who consumed homemade peanut oil and not.

Variable	Total (*N* = 1611)	Homemade peanut oil (*N* = 1316)	Non-homemade peanut oil (*N* = 295)	*p*
**Behavioral factors**				
Physical exercise during pregnancy				0.372
Yes	1373 (85.2)	1127 (85.6)	246 (83.4)	
No	238 (14.8)	189 (14.4)	49 (16.6)	
Drinking				1.000
Yes	15 (0.9)	12 (0.9)	3 (1.0)	
No	1596 (99.1)	1304 (99.1)	292 (99.0)	
Smoking				0.127
Yes	13 (0.8)	8 (0.6)	5 (1.7)	
No	1598 (99.2)	1308 (99.4)	290 (98.3)	
Passive smoking				0.861
No	645 (40.0)	527 (40.0)	118 (40.0)	
Work place or home	710 (44.1)	577 (43.9)	133 (45.1)	
Work place and home	256 (15.9)	212 (16.1)	44 (14.9)	
**Obstetric information**				
History of PB				0.432
Yes	82 (5.1)	56 (4.3)	9 (3.1)	
No	1529 (94.9)	1260 (95.7)	286 (96.9)	
History of LBW				0.721
Yes	46 (2.9)	39 (3.0)	7 (2.4)	
No	1565 (97.1)	1277 (97.0)	288 (97.6)	
History of abortion				0.399
Yes	264 (16.4)	221 (16.8)	43 (14.6)	
No	1347 (83.6)	1095 (83.2)	252 (85.4)	
History of HBV				0.056
Yes	82 (5.1)	74 (5.6)	8 (2.7)	
No	1529 (94.9)	1242 (94.4)	287 (97.3)	
Parity				<0.001
1	695 (43.2)	158 (53.6)	537 (40.8)	
2	558 (34.6)	96 (32.5)	462 (35.1)	
≥3	358 (22.2)	41 (13.9)	317 (24.1)	
Pre-pregnancy BMI (kg/m^2^)				0.017
Underweight (<18.5)	317 (19.7)	256 (19.5)	61 (20.7)	
Normal (18.5–22.9)	980 (60.8)	786 (59.7)	194 (65.8)	
Overweight or obese (≥23)	314 (19.5)	274 (20.8)	40 (13.5)	
Pregnancy complications				0.636
No	1350 (83.8)	1106 (84.0)	244 (82.7)	
Yes	261 (16.2)	210 (16.0)	51 (17.3)	
Folic supplementation				0.013
Yes	1471 (91.3)	1213 (92.2)	258 (87.5)	
No	140 (8.7)	103 (7.8)	37 (12.5)	
Calcium supplementation				0.491
Yes	1261 (78.2)	1035 (78.6)	226 (76.6)	
No	350 (21.8)	281 (21.4)	69 (23.4)	
Iron supplementation				0.110
Yes	609 (37.8)	510 (38.8)	99 (33.6)	
No	1002 (62.2)	806 (61.2)	196 (66.4)	
Vitamin D supplementation				0.562
No	1463 (90.8)	1192 (90.6)	271 (91.9)	
Yes	148 (9.2)	124 (9.4)	24 (8.1)	
Medicine intake				0.360
No	1410 (87.5)	1157 (87.9)	253 (85.8)	
Yes	201 (12.5)	159 (12.1)	42 (14.2)	

Abbreviations: PB, preterm birth; LBW, low birth weight; BMI, body mass index; HBV, *hepatitis B virus*.

The birth outcomes of study women who consumed or did not consume homemade peanut oil are presented in Table 3. Of 1611 newborns, the average gestational age at birth was 38.2 ± 1.8 weeks in the homemade peanut oil group and 38.5 ± 1.6 weeks in the non-homemade peanut oil group. Mode of delivery, newborn sex and average birth weight were not significantly different between the two groups. A significantly higher rate of LBW was found in the homemade peanut oil group, at 10.6% vs 5.8% in the non-homemade peanut oil group (*p* = 0.015). The rate of PB was 10.7% in the homemade peanut oil group compared with 6.8% in the non-homemade peanut oil group, but the difference was not significant.

**Table 3.** Birth outcomes of study women who currently consumed and did not consume homemade peanut oil.

Variable	Total (*N* = 1611)	Homemade peanut oil (*N* = 1316)	Non-homemade peanut oil (*N* = 295)	*p*
Birth gestation age (weeks)	38.3 ± 1.8	38.2 ± 1.8	38.5 ± 1.6	0.005
Mode of delivery				0.065
Vaginal delivery	1127 (70.0)	907 (68.9)	220 (74.6)	
Cesarean section or assisted vaginal delivery	484 (30.0)	409 (31.1)	75 (25.4)	
Newborn sex				0.377
Male	832 (51.6)	687 (52.2)	145 (49.2)	
Female	779 (48.4)	629 (47.8)	150 (50.8)	
Birth weight (gm)	3071.8 ± 508.2	3068.8 ± 518.1	3085.1 ± 462.2	0.593
Low birth weight				0.015
Yes	157 (9.7)	140 (10.6)	17 (5.8)	
No	1454 (90.3)	1176 (89.4)	278 (94.2)	
Preterm birth				0.054
Yes	161 (10.0)	141 (10.7)	20 (6.8)	
No	1450 (90.0)	1175 (89.3)	275 (93.2)	

**Figure 2.**
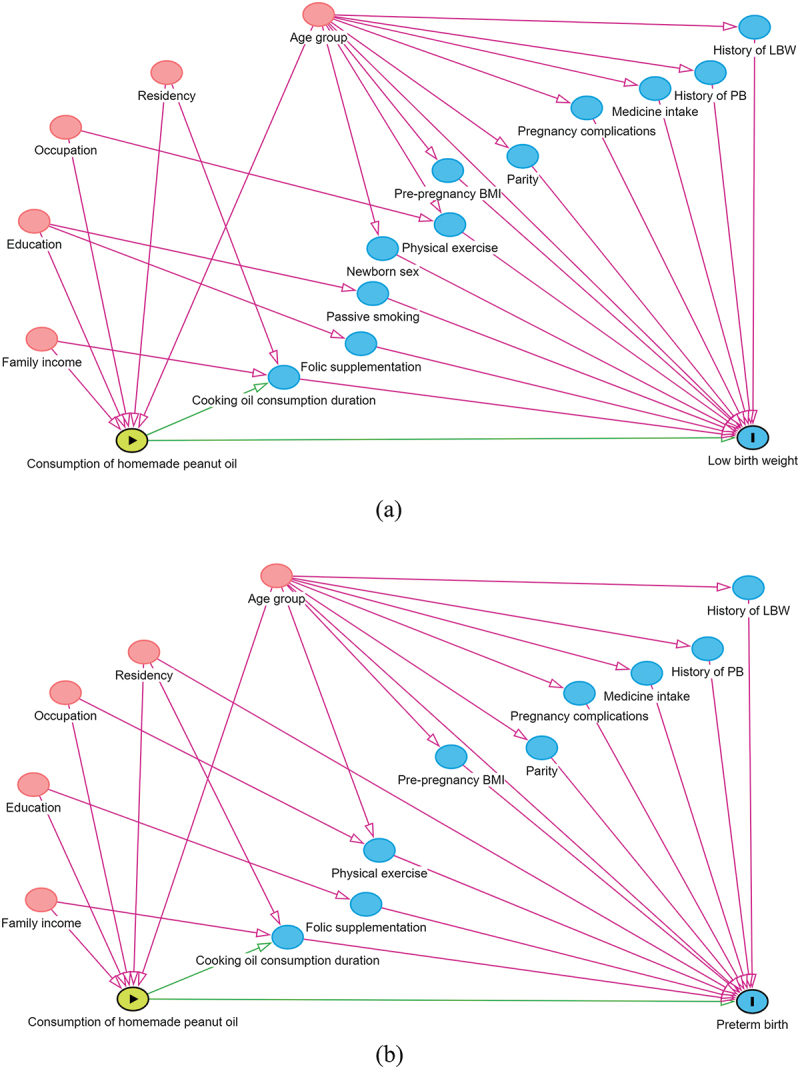
The DAGs showing the related confounders of homemade peanut oil and LBW (a) and PB (b).

All factors associated with homemade peanut oil as the main exposure of interest and the LBW and PB outcomes are shown in the DAG diagrams in Figure 2(a) and (b), respectively. The direct and total effects of homemade peanut oil consumption on LBW and PB are shown in Table 4. After adjusting for confounders under the DAG model, the odds of having LBW (aOR_total_ 1.8, 95% CI 1.1–3.1 and aOR_direct_ 1.9, 95% CI 1.1–3.2) and PB (aOR_total_ 1.7, 95% CI 1.0–2.8 and aOR_direct_ 1.8, 95% CI 1.1–3.0) were higher in women who consumed homemade peanut oil in both the direct and total effect models, respectively.

DSGs showing different relationship pathways of homemade peanut oil consumption with low birth weight (LBW) or preterm birth (PB) are adjusted by other variables (created in a browser-based environment, DAGitty: https://www.dagitty.net). In the DAGs, each circle represents a node of one variable (blue circle with vertical line = main outcome; green circle with triangle = main exposure; blank blue circle = mediating circle or ancestor of outcome; blank orange circle = confounding or biasing variable). The green line with arrows indicates the only unidirectional causal pathway that comes from main exposure to main outcome; orange and pink lines with arrows indicate unidirectional causal pathways. DAG = directed acyclic graph.

**Table 4.** Direct and total effects of exposure to homemade peanut oil consumption on LBW and PB.

Main outcome	Variable	Adjusted variables	Effect	cOR	aOR (95% CI)
LBW	Consumption of homemade peanut oil	Age group, cooking oil consumption duration, education, occupation	Direct	1.9 (1.2 - 3.3)	1.9 (1.1 - 3.2)
		Age group, education, occupation, residency, family income	Total		1.8 (1.1 - 3.1)
PB	Consumption of homemade peanut oil	Age group, cooking oil consumption duration, education, occupation, residency	Direct	1.6 (1.0 - 2.7)	1.8 (1.1 - 3.0)
		Age group, education, occupation, residency, family income	Total		1.7 (1.0 - 2.8)

Abbreviations: LBW, low birth weight; PB, preterm birth; cOR, crude odds ratio; aOR, adjusted odds ratio; CI, confidence interval.

**Figure 3.**
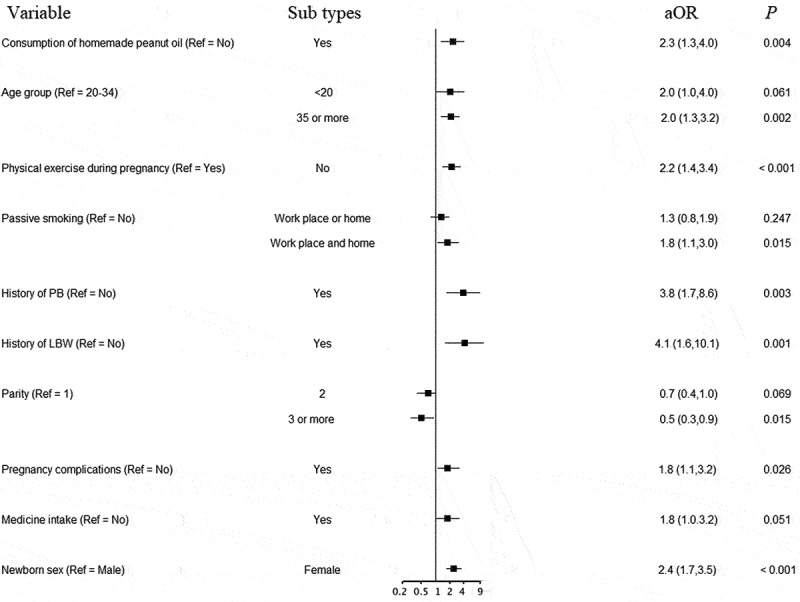
Factors associated with low birth weight using a multiple logistic regression model.

**Figure 4.**
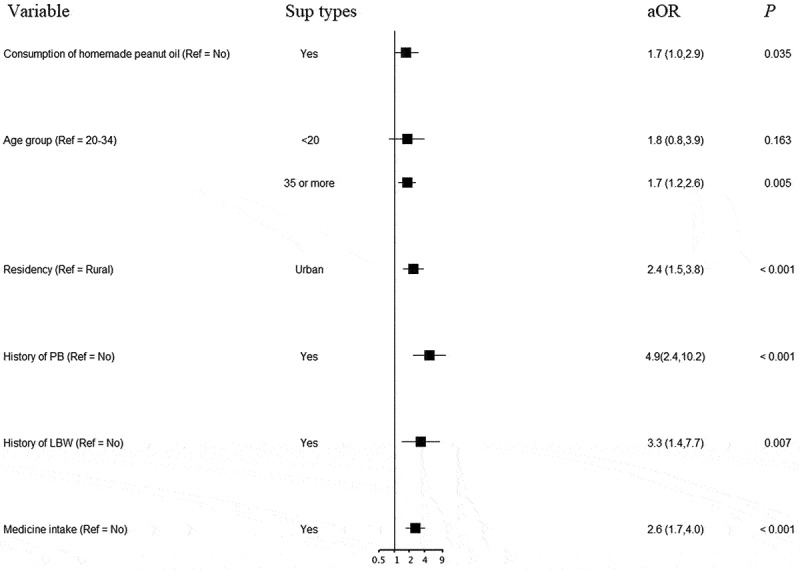
Factors associated with preterm using a multiple logistic regression model.

Other independent risk factors for LBW and PB analyzed using multiple logistic regression models are shown in Figures 3 and 4. Apart from consumption of homemade peanut oil, age over 35 years, having no physical exercise during pregnancy, exposure to passive smoking in both workplace and home, having a history of PB or LBW, having pregnancy complications, and female newborn were significantly associated with LBW (Figure 3). The odds of having PB were higher in women aged over 35 years, urban residency, having a history of PB or LBW, and medicine intake during pregnancy in addition to consuming homemade peanut oil (Figure 4).

## Discussion

The rates of LBW and PB in pregnant women who consumed homemade peanut oil during pregnancy were almost double compared to those who consumed non-homemade peanut oil in our study in a county in Southwestern China. Advanced maternal age and history of LBW or PB were associated with higher rates of both LBW and PB. No physical exercise during pregnancy, passive smoking, pregnancy complications, and female newborn were associated with LBW, while urban residency and medicine intake during pregnancy were additionally associated with risk for PB.

To our knowledge, there have been no other studies to date examining the association between risk of LBW or PB and consumption of homemade peanut oil as in our study. A previous study reported lower birth weight and gestational age at birth in Guangxi, where consumption of homemade peanut oil is prevalent, than in other areas having low homemade peanut oil consumption, which implied that homemade peanut oil consumption during pregnancy may impact the birth weight and gestation age at birth [25]. We found a similar effect of homemade peanut oil consumption on LBW and PB regardless of confounding factors or mediating adjustments that were age group, education, occupation, residency, and family income, indicating that homemade peanut oil consumption was almost certainly related to the occurrence of LBW and PB. Scientific support of this finding can be explained through the contamination of AFs in homemade peanut oil, as two previous studies conducted in Guangxi reported high AFs in homemade peanut oil [8,9]. Guangxi is one of the provinces for homemade peanut oil contamination by AFB1 in China [26], with the finding double the AFB1 contamination in peanut samples than peanut oil samples. Another previous study found that fetuses were at a higher risk of AFs than adults when exposed to AFs [27].

Apart from homemade peanut oil consumption, it was not surprising that maternal age, complications during pregnancy, medication intake, newborn sex, parity, and residency were associated with LBW and PB in our study. Increasing LBW and PB in advanced maternal age was consistent with previous studies for LBW [28–30] and for PB [31–34]. However, our finding was different from a study from China in which no significant association between maternal age and LBW was found [17]. It was not surprising that women with a history of PB or LBW had higher odds of having LBW and PB, which was consistent with previous studies [30,32,35,36]. This may be explained by chronic exposure to unknown factors for LBW and PB.

We found that LBW was higher in pregnant women who had further complications or were taking medication during pregnancy, as was also found in previous studies [28,35,37]. Likewise, female newborns had significantly higher odds of having LBW than males [17], and there were lower odds of having LBW in women with higher parity [38]. The odds of having PB in women living in an urban area were higher than in women in a rural area, which was opposite to the results of a recent systematic review study [35]. This could be due to other unknown factors which are different between rural and urban areas.

Those who reported no physical exercise during pregnancy had higher odds of having an LBW infant, which was consistent with a previous study in China [39] but different from the conclusion of a systematic review [40], which might be because the characteristics of women and exercise measurement were not the same. The association between passive smoking and LBW in our study was consistent with a previous study [39].

Our study highlights the relationship between homemade peanut oil consumption and LBW and PB using a prospective cohort study with a relatively large sample. Even though there have been many studies examining AF exposure, including diet and food exposure [41–46], we were unable to find any publications examining the effect of homemade peanut oil consumption on pregnant women and their newborn LBW and PB outcomes. In addition, our study not only used DAGs to adjust for confounders and mediators but also explored independent, related factors, which can provide more information for governments to control the risk of AFs exposure.

There were some limitations in the study. First, the levels of oil consumption during pregnancy were obtained through a questionnaire, which may have had recall bias. However, this bias would be minimal since the duration of time following the consumption ranged from 7 to 10 months and the lifestyles of the study group tend to be static, particularly concerning things such as the use of cooking oils. Second, the histories of behavioral factors relied on women’s self-reports. Third, we did not collect the cause of PB, therefore we could not identify whether homemade peanut oil consumption can be significantly related to spontaneous or iatrogenic cause of PB. Fourth, the sample size was calculated for the outcome based on exposure to homemade peanut oil, not other independent factors such as socioeconomic or pre-pregnancy BMI. Fifth, our study did not assess the dietary intake for calculating calorie and nutrient assessments during pregnancy. Finally, the levels of AFs in homemade peanut oil samples and homemade peanut oil consumption were not presented in this cohort study; however, their findings from a subset of the study were measured and submitted elsewhere.

## Conclusions

Homemade peanut oil consumption was associated with increased risk of LBW and PB, in addition to advanced age, adverse obstetric histories, and health risk behaviors during pregnancy in a county in Southwestern China. Governments should pay more attention to the consumption of homemade peanut oil during pregnancy. Close monitoring and appropriate counseling are required for high-risk pregnant women with LBW and PB. The hypothesis that AFs in homemade peanut oil are associated with LBW and PB should be tested.

## Paper context

Homemade peanut oil is commonly contaminated by aflatoxin in Southwestern China. However, there were few studies on adverse pregnancy outcomes in pregnant women who consumed homemade peanut oil during pregnancy. Our study found that homemade peanut oil consumption was one of the significant risk factors of low birth weight and preterm birth. The findings of associated risk factors should be informed to the community, and precautionary policies for the protection of aflatoxin exposure during pregnancy are needed.
